# Transition to remote/hybrid learning during the COVID-19 pandemic among Saudi students of the College of Applied Medical Sciences: a cross-sectional study

**DOI:** 10.3389/fmed.2023.1257589

**Published:** 2023-08-22

**Authors:** Khalid M. Alshamrani, Enas M. Ghulam, Maryam Alattas, Haneen Aljaddani, Montaha Alhakami, Ziyad F. Al Nufaiei, Majid S. Althaqafy

**Affiliations:** ^1^College of Applied Medical Sciences, King Saud bin Abdulaziz University for Health Sciences, Jeddah, Saudi Arabia; ^2^King Abdullah International Medical Research Center, Jeddah, Saudi Arabia; ^3^Ministry of the National Guard - Health Affairs, Jeddah, Saudi Arabia; ^4^College of Science and Health Professions, King Saud bin Abdulaziz University for Health Sciences, Jeddah, Saudi Arabia

**Keywords:** COVID-19, e-learning, hybrid model, health education, Kingdom of Saudi Arabia

## Abstract

**Background:**

The novel Coronavirus Disease 2019 (COVID-19) pandemic has presented unparalleled and unique stressors and challenges to the field of applied health sciences education. This study explored how the College of Applied Medical Sciences (COAMS) Saudi students perceive the transition to remote/ hybrid learning during the COVID-19 pandemic.

**Methods:**

A cross-sectional exploratory investigation was carried out during the months of February and March in the year 2023 among 196- COAMS Saudi students, using the 48-item previously developed and validated questionnaire, and with a non-probability convenient sampling technique. Descriptive statistics were generated for participants’ demographics, and for each questionnaire item and statistical analysis was performed using chi-square test.

**Results:**

Out of the 283 undergraduates who have enrolled in COAMS, a total of 196 students have agreed to participate in the study with an overall response rate of 69.3%. Over 70% of COAMS students were satisfied and engaged in their on-site coursework. Nevertheless, questionnaire data indicates that their satisfaction and level of engagement diminished following the shift to remote learning. More than 62% of COAMS students were satisfied with their instructors’ instructional and assessment strategies during on-site coursework, but such perceptions have decreased with remote instruction. Hybrid learning can be beneficial and effective in improving the performance and learning experience of COAMS students. As compared to female students, COAMS male students were more satisfied with remote learning because it met their needs (*p* = 0.017).

**Conclusion:**

Remote classrooms have lower attendance and interest than on-site classes. Despite lower satisfaction levels in online courses, hybrid learning was viewed favourably by COAMS students. Higher educational institutions should develop plans to increase student involvement, improve academic integrity, and assess the effect of the pandemic on undergraduate education on a regular basis. By incorporating these measures, educational institutions can enhance and support the remote learning experience for their students.

## Introduction

During the novel Coronavirus Disease 2019 (COVID-19) pandemic, the world encountered numerous challenges in preventing the spread of the virus. These challenges, which included social distancing and quarantine, had a significant impact on the lifestyles, physical and psychological health, and well-being of college students (1–3). Many nations across the world have declared a state of emergency in response to the pandemic, resulting in a transformation of the educational system from traditional face-to-face learning to remote learning to ensure the continuity of students’ academic pursuits (4).

The rapidly changing learning environment has a significant impact on students. Furthermore, the quality of online education varies among faculty (5), with teamwork, technology, a shared interest, and the nature of the task are all crucial components of successful distance learning (6). Universities have modified their pedagogical approaches amid the epidemic, implementing flipped classes (7), peer education (8), and innovative ways of delivering course material to motivate and encourage students (7). Additionally, higher-educational institutions may face challenges in accurately assessing students’ academic performance due to the prompt and easy access to information during examinations (9).

The COVID-19 pandemic has brought the educational divide into focus, with a disproportionate impact on students in rural and low-income communities, highlighting a significant educational and socioeconomic disparities (10). Furthermore, various technology platforms have been explored including Microsoft Teams, Zoom, Canvas, Google Hangouts, and Webex (9, 11). Both faculty and students have provided valuable insights into the strengths and weaknesses of various platforms, including which are most user-friendly and easy to use (12). In general, faculty have shown unfavorable attitudes toward remote instruction, particularly in courses that rely on fieldwork and labs. However, certain remote teaching methods have been deemed effective, such as asynchronous instruction that entails minimal interaction with the instructor or peers (9, 13). Anatomy instructors in Singapore have expressed their support for Zoom and narrated Powerpoint presentations for teaching pharynx, larynx, and ear anatomy to medical students (14). Even though many faculty recognize the benefits of remote learning, they acknowledge that it cannot fully replace the value of in-person instruction. Kuwait’s public institutions’ spring semester has been postponed until August 2020 due to the belief that online education would not replace the traditional classroom environment (15). The trend towards remote teaching and learning may persist for long time, and it has led to advances in pedagogical practices (9, 12, 16). Although the convenience of remote learning is well-acknowledged, two-thirds of medical students who participated in a Duke University survey preferred to return to one-on-one teaching (17). Additionally, students have described how the pandemic has affected their social interactions, physical activity levels, overall stress levels, as well as how these alterations have changed their ability to remain productive (18, 19). Another growing concern is the mental health of students, with stress, anxiety, and burnout being reported by university students during the COVID-19 pandemic (2, 20).

Hybrid learning is a pedagogical approach that combines conventional in-person instruction with online learning systems (21, 22). A hybrid educational system combines online courses with practical activities in laboratories which allow students to meet their teachers and classmates. In higher education institutions, hybrid learning has been found to motivate students, manage high dropout rates, and promote a sense of safety (23). At the beginning of the hybrid education system period, educators use in-person interactions to give students an overview of online tools, how to get help, and how assessments will be done (24).

The engagement of students is strongly tied to the effectiveness of teaching, with the characteristics and qualities of the instructor serving as crucial factors in enhancing student engagement and satisfaction (25); therefore, highly effective instructors are more likely to engage their students and develop an inclusive teaching and learning environment (i.e., on-site, remote, or hybrid), which will result in higher academic performance, expectations, and student satisfaction (26–28). Student engagement refers to the level of energy and effort that students put into their learning community, which can be observed through various behavioral, cognitive, and affective indicators, and is influenced by a variety of factors, both internal and external, such as the intricate interplay of relationships, learning activities, and the learning environment (29). Student engagement has three dimensions, behavioral, cognitive, and affective, with several engagement indicators alongside disengagement indicators in each dimension (29–36).

Behavioral engagement encompasses indicators related to a student’s physical actions, such as attending, participating, getting involved, and interacting in class activities (28, 34–36). Additional indicators of behavioral engagement include attentiveness, achievement, confidence, and study habits (29). In contrast, behavioral disengagement is indicated by absence, half-heartedness, lack of focus, inattentiveness, distraction, poor conduct, and giving up (29). For instance, a behaviorally engaged students would attend the applied health sciences’ sessions or workshops, avoid distractions, and actively participate in note-taking, group discussions, or audience response (28, 30, 32, 33).

Affective engagement encompasses indicators related to a student’s positive emotions, attitude, and interaction in response to the teacher, peers, and the education environment (28, 29, 33, 34, 36, 37). Additional indicators of affective engagement include enjoyment, interest, motivation, and enthusiasm (29, 38, 39). In contrast, affective disengagement is indicated by negative emotions including frustration, disappointment, worry, anxiety, boredom, and disinterest (29, 33, 35). For instance, a student who is emotionally engaged would find the educational session enjoyable and would not be disinterested and preoccupied with the passage of time (28).

Cognitive engagement entails possessing the ability to actively participate in self-regulated learning and holding a deep appreciation for the significance of learning (28, 35–37). Additional indicators of cognitive engagement include learning from peers, deep learning, positive self-perception, the perceived relevance of material to the student’s experience, and critical thinking (28, 29). In contrast, cognitive disengagement is indicated by opposition, rejection, feeling pressured, unwillingness, avoidance, and feeling overwhelmed (29). For instance, a student who possesses a high level of cognitive engagement would be able to recognize the practicality of the course material in their future endeavors and would be driven to acquire more knowledge about the subject matter, even beyond the confines of the classroom (28, 33).

The College of Applied Medical Sciences (COAMS) at King Saud bin Abdulaziz University for Health Sciences (KSAU-HS) in the Kingdom of Saudi Arabia (KSA) (40), is comprised of eight academic programs and a four-year curriculum study plan that offers preprofessional science and health courses to students during their first 2 years of study. During the third and fourth years of the study, students begin their professional studies with various field/clinical experiences that provide hands-on training. A combination of face-to-face, online and hybrid learning systems is used by COAMS programs to deliver their curriculum, which is designed to be dynamic with a variety of instructional methods (41). As remote and hybrid learning is expected to become a fabric of health professions education in the future, a comprehensive understanding of COAMS students’ attitude toward and how they perceive face-to-face, remote and hybrid learning environments is essential to determining the benefits and drawbacks of COAMS curriculum delivery models and thus improving the health professions educational system in pandemics and emergencies like COVID-19. The underlying theoretical framework assumes that COAMS students have concerns that require attention, as their perceptions of faculty preparedness, student effort, engagement, needs, and ethical behavior during remote and hybrid instruction are essential for COAMS faculty and administration to effectively adapt curricular change *via* the “Concerns-Based Adoption Model (CBAM)” (42), which is a theoretical framework developed to help leaders and faculty in understanding, guiding, and overseeing the complex process of change in education. In this study, the students’ concerns were related to the conceptualization, teaching and adoption of remote and hybrid learning in the COAMS educational system.

Students’ experience and perspectives regarding face-to-face, online and hybrid educational systems/environments hold immense significance for the advancement of education in the future, as well as for the development of flexible courses by faculty. The literature on students’ perspectives in these environments is well documented (6, 12, 17, 21–24, 43–53), and it is likely that we will witness an increase in interest in this area in the coming years, as educational institutions strive to ensure well-designed learning experiences, given that natural disasters and crises may once again disrupt face-to-face learning. Research studies have been conducted recently among undergraduate students to better understand the various aspects of transitioning to remote or hybrid instruction during COVID-19. These surveys sought to gather information on the student experience as they transitioned to remote learning (12, 24, 45, 46, 52), the students’ reactions to the modifications made to courses (45), the pros and cons of remote instruction (46), changes in student participation patterns (47), as well as the course structures and instructor strategies that increased or decreased engagement during the transition (6, 47). Other recent studies also explored students’ perceptions of engagement and the factors that influenced engagement in online classes (6, 50). Additionally, recent studies also examined students’ experiences and opinions about hybrid learning and its challenges and opportunities (22, 51, 53). Similarly, our study considers COAMS Saudi students’ perceptions for the purpose of gathering information to generally understand the student experience of the transition to remote/ hybrid learning during COVID-19. The current study investigates how 196 COAMS Saudi students perceive the transition to remote/ hybrid learning during COVID-19, and aims to answer the following questions:

1. How has the transition from on-site to remote learning affected the attendance and completion of coursework by COAMS students?
2. Would the COAMS students be more interested, engaged, and satisfied with on-site versus remote learning, and how effective are the instructional and assessment methods of their instructors?
3. Is hybrid learning beneficial and effective in improving performance and learning outcomes for COAMS students?

COAMS faculty will be able to improve the effectiveness of teaching in their courses and the overall learning environment by addressing these questions, which encompass the three above-mentioned dimensions of student engagement, including multiple aspects/facets of each dimension. To the best of our knowledge, no study has ever explored the strengths and limitations of remote / hybrid educational systems among Saudi undergraduate applied medical sciences students.”

## Methods

### Participants, setting, sampling technique and procedure

A cross-sectional exploratory investigation was carried out during the months of February and March in the year 2023 among students of the COAMS at KSAU-HS, Jeddah campus, KSA. Utilizing a non-probability convenient sampling technique (i.e., strategy where participants are selected for this situation based on their accessibility and/or proximity to the research) (54), the entire population consisting of 283 male and female undergraduate students in their third and fourth years was invited to participate in this study *via* WhatsApp messaging and email invitation. The online questionnaire was administered using electronic survey tool (i.e., Google form).

### Data instruments

The participants were instructed to complete two questionnaires that had been previously utilized. The content validity of the questionnaires was assumed since we relied on previously published conceptual frameworks and prior instruments. This is in accordance with Messick’s framework for validity of psychometric assessment (55), and as suggested by “Cook, & Beckman, 2006” (56). The first questionnaire was the self-constructed six dimensions/subscales questionnaire, which consisted of 42 items, as developed by Parker et al. (12). The second questionnaire was the blended or hybrid learning-related 6 items questionnaire, created by Al-Fodeh et al. (51). All closed-response items were measured using a five-point Likert scale, with response options ranging from strongly disagree (1), disagree (2), neutral (3), agree (4) to strongly agree (5). For the purposes of reliability analysis, those items where agreement indicated negative behavior, such as cheating, were reverse coded.

The 42-item questionnaire developed by Parker et al. was comprised of six distinct dimensions/subscales. These subscales included: general perceptions on in-person learning prior to COVID-19 (9 items); general perceptions on remote learning prior to COVID-19 (9 items); initial perceptions regarding remote learning (7 items); perceptions of the students regarding their effort and engagement while taking online classes (8 items); students’ perceptions concerning their needs, behavior, and perception of effort while taking online classes (4 items); and ethical behavior (5 items).

### Ethical consideration

King Abdullah International Medical Research Center ethics committee approved this study (Study Number: SP22J/123/08).” Participation was completely voluntary, and prior to completing the questionnaire, written informed consent was acquired. Anonymity and confidentiality were strictly upheld throughout the research process. The Microsoft Excel file, which was exported from the electronic survey tool, was encrypted with a password, and did not reveal any subject identification attributes. This research did not receive any specific grant from funding agencies in the public, commercial, or not-for-profit sectors. We confirm that a written informed consent was obtained from the study participants and that the guidelines outlined in the Declaration of Helsinki were followed.

### Statistical analyses

The statistical analysis was comprised of a three-step process. First, frequencies, and percentages were generated for the participants’ demographics encompassing gender, academic year, and academic program. Additionally, descriptive statistics (i.e., counts, percentages “%,” median “Mdn,” mean “μ,” and standard deviation “SD”), were generated for each questionnaire item. Second, the internal consistency reliability of the questionnaire’s seven dimensions/subscales was evaluated using Cronbach’s alpha test. Third, chi-square test of independence was used to examine differences in questionnaire responses for demographic variables (i.e., gender, academic year). All analyses were carried out using JMP^®^ Software (JMP^®^, Version 16. SAS Institute Inc., Cary, NC, 1989–2023) and a statistical significance level of 0.05 was employed.

## Results

### Demographics

Descriptive statistics pertaining to the demographics of COAMS participated students are shown in Table 1. Out of the 283 undergraduates who have enrolled in COAMS, a total of 196 students have agreed to participate in the study with an overall response rate of 69.3%. Of these participants, 102 (52%) are female and 94 (48%) are male. Moreover, 106 (54.1%) are in their 3rd year while 90 (45.9%) are in their 4th year. Of the 196 participating students, 14 (7.1%) were enrolled in the anesthesia technology program, 11 (5.6%) in the clinical laboratory science program, 13 (6.6%) in the clinical nutrition program, 15 (7.7%) in the echocardiography cardiovascular technology program, 34 (17.4%) in the emergency medical services program, 29 (14.8%) in the occupational therapy program, 27 (13.8%) in the radiological sciences program, and 53 (27%) in the respiratory therapy program.

**Table 1:** Descriptive statistics of the studied sample.

Variable		Participants *n* = 196	
		Number of responses	Percentage of responses (%)
Female		102	(52%)
Male		94	(48%)
3rd year		106	(54.1%)
4th year		90	(45.9%)
**Academic program**	**Gender**		
1. Anesthesia Technology	Male only	14	(7.1%)
2. Clinical Laboratory Science	Female only	11	(5.6%)
3. Clinical Nutrition	Female only	13	(6.6%)
4. Echocardiography Cardiovascular Technology	Female only	15	(7.7%)
5. Emergency Medical Services	Male & Female	34	(17.4%)
6. Occupational Therapy	Male & Female	29	(14.8%)
7. Radiological Sciences	Male & Female	27	(13.8%)
8. Respiratory Therapy	Male & Female	53	(27%)

$\text{Percentage of Responses}\;\left(\%\right)=\frac{\text{Number of Responses}}{196}\times 100$.

### Questionnaire internal consistency findings

Table 2 shows the statistical data of internal consistencies (Cronbach’s alpha) for the seven dimensions/subscales of the questionnaire. The results from the scales show acceptable, good, and excellent internal consistency reliability both overall and by subscale. The overall internal consistency reliability for the 48 items was α = 0.915. The subscales also showed high internal consistency reliability: general perceptions on in-person learning prior to COVID-19 (9 items, α = 0.731), general perceptions on remote learning prior to COVID-19 (9 items, α = 0.744), initial perceptions regarding remote learning (7 items, α = 0.833), Students’ perceptions of their effort and engagement (8 items, α = 0.785), students’ needs, behavior, and perception of effort (4 items, α = 0.743), student ethics (5 items, α = 0.800), and general perceptions of hybrid learning (6 items, α = 0.928).

**Table 2:** Internal consistencies (Cronbach’s alpha) statistics.

Dimensions/Subscales	Number of items	Cronbach’s alpha Score
1. General Perceptions of Learning: On-site	9	0.731
2. General Perceptions of Learning: Remote	9	0.744
3. Initial Perceptions about Remote Learning	7	0.833
4. Student Effort and Engagement	8	0.785
5. Student Needs, Behavior, and Perception of Effort	4	0.743
6. Student Ethics	5	0.800
7. General Perceptions of Learning: Hybrid	6	0.928
Total	48	0.914

### Questionnaire responses findings

Tables 3–9 present the outcomes of the Likert-response questionnaire items from all participants. In order to simplify interpretation, percentages and counts from agree and strongly agree options as well as disagree and strongly disagree options were contextually combined for all items. Table 3, in particular, shows the general perception of “On-Site” learning among COAMS students prior to COVID-19. When it comes to their personal conduct, the majority of COAMS students expressed agreement and strong agreement towards attending on-site classes (*n* = 151, 77.1%), completing on-site coursework (*n* = 152, 77.6%), being engaged in on-site courses (*n* = 127, 64.8%), and being satisfied with on-site coursework (*n* = 138, 70.4%). On the other hand, only 36.2% (*n* = 71) of COAMS students agreed and strongly agreed that they were challenged by on-site coursework; whereas 35.7% (*n* = 70) were neutral, and 28% (*n* = 55) disagreed and strongly disagreed. Furthermore, the vast majority of COAMS students disagreed and strongly disagreed (*n* = 159, 81.1%) with cheating during on-site courses. When COAMS students were asked to provide their opinions regarding their instructors, a large proportion agreed and strongly agreed that their professors used effective instructional strategies during on-site coursework (*n* = 123, 62.8%), used effective assessment strategies (*n* = 127, 64.8%), and were engaged with their instruction (*n* = 141, 71.9%). Table 4 shows identical set of questions regarding the general perception of “Remote” learning among COAMS students prior to COVID-19. When it pertains to their personal conduct, a substantial proportion of COAMS students expressed agreement and strong agreement towards attending remote classes (*n* = 126, 64.3%), and completing remote coursework (*n* = 145, 74%). In contrast, approximately half of COAMS students expressed agreement and strong agreement regarding their engagement in remote courses (*n* = 95, 48.4%), and satisfaction with remote coursework (*n* = 109, 55.6%). Furthermore, only 40.3% (*n* = 79) of COAMS students agreed and strongly agreed that they were challenged by remote coursework; whereas 28.6% (*n* = 56) were neutral, and 31.1% (*n* = 61) disagreed and strongly disagreed. Moreover, a larger proportion of COAMS students disagreed and strongly disagreed (*n* = 121, 61.7%) with cheating during remote courses. When COAMS students were asked to provide their opinions regarding their instructors, approximately half agreed and strongly agreed that their professors used effective instructional strategies during remote coursework (*n* = 95, 48.5%), used effective assessment strategies (*n* = 99, 50.5%), and were engaged with their instruction (*n* = 103, 52.6%). Tables 5–8 address specific details regarding the remote learning experience. On average, 65.5% (*n* = 129) of COAMS students have expressed agreement and strong agreement with regards to the adequacy of their internet access and internet speed for remote instruction purposes, as shown in Table 5. Additionally, more than half of the COAMS students agreed and strongly agreed that instructors’ instructional methods (*n* = 115, 58.7%), and assessment methods (*n* = 106, 54%) translated well into remote instruction. Also, 40.8% of COAMS students (*n* = 80) have expressed agreement and strong agreement that their professors worked harder to provide remote instruction than on-site instruction, while 50.6% (*n* = 99) have expressed agreement and strong agreement that their professors worked harder to provide on-site instruction than remote instruction. Moreover, approximately half of COAMS students expressed agreement and strong agreement regarding their satisfaction with remote instruction (*n* = 99, 50.5%).

**Table 3:** COAMS students’ general perceptions of “On-site” learning prior to COVID-19.

	*n* (%)					Mdn	μ	SD
Item	Strongly disagree Code = 1	Disagree Code = 2	Neutral Code = 3	Agree Code = 4	Strongly agree Code = 5	Summary statistics		
1. I attended the vast majority of on-site classes.	6 (3.1%)	10 (5.1%)	29 (14.8%)	37 (18.9%)	114 (58.2%)	5	4.24	1.07
2. I was challenged by my on-site course work.	23 (11.7%)	32 (16.3%)	70 (35.7%)	48 (24.5%)	23 (11.7%)	3	3.08	1.16
3. I completed the vast majority of my on-site course work.	4 (2%)	7 (3.6%)	33 (16.8%)	48 (24.5%)	104 (53.1%)	5	4.22	0.99
4. I was engaged in my on-site courses.	9 (4.6%)	13 (6.6%)	47 (24.0%)	57 (29.1%)	70 (35.7%)	4	3.85	1.12
5. I was satisfied with my on-site course work.	8 (4.1%)	15 (7.7%)	35 (17.9%)	60 (30.6%)	78 (39.8%)	4	3.94	1.12
*Item*	*Strongly disagree Code = 5*	*Disagree Code = 4*	*Neutral Code = 3*	*Agree Code = 2*	*Strongly agree Code = 1*			
6. I cheated during my on-site courses.*	138 (70.4%)	21 (10.7%)	17 (8.7%)	10 (5.1%)	10 (5.1%)	1	1.64	1.15
*Item*	*Strongly disagree Code = 1*	*Disagree Code = 2*	*Neutral Code = 3*	*Agree Code = 4*	*Strongly agree Code = 5*			
7. My professors used effective instructional strategies during my on-site course work.	7 (3.6%)	20 (10.2%)	46 (23.5%)	57 (29.1%)	66 (33.7%)	4	3.79	1.12
8. My professors used effective assessment strategies during my on-site course work.	12 (6.1%)	16 (8.2%)	41 (20.9%)	63 (32.1%)	64 (32.7%)	4	3.77	1.17
9. My professors were engaged in my on-site course work.	6 (3.1%)	13 (6.6%)	36 (18.4%)	61 (31.1%)	80 (40.8%)	4	4	1.07

COAMS, College of Applied Medical Sciences; Mdn, Median; μ, Mean; and SD, Standard deviation. $\text{Percentage of Responses}\;\left(\%\right)=\frac{\text{Number of Responses}}{196}\times 100.$ *Items where agreement indicated negative behavior, such as cheating, were reverse coded for the purposes of reliability analysis.

**Table 4:** COAMS students’ general perceptions of “Remote” learning prior to COVID-19.

	n (%)					Mdn	μ	SD
Item	Strongly disagree Code = 1	Disagree Code = 2	Neutral Code = 3	Agree Code = 4	Strongly agree Code = 5	Summary statistics		
1. I attended the vast majority of live, remote classes.	7 (3.6%)	19 (9.7%)	44 (22.4%)	45 (23.0%)	81 (41.3%)	4	3.89	1.16
2. I was challenged by my remote course work.	26 (13.3%)	35 (17.9%)	56 (28.6%)	47 (24.0%)	32 (16.3%)	3	3.12	1.26
3. I completed the vast majority of my remote course work.	7 (3.6%)	12 (6.1%)	32 (16.3%)	54 (27.6%)	91 (46.4%)	4	4.07	1.09
4. I was engaged in my remote courses.	14 (7.1%)	33 (16.8%)	54 (27.6%)	43 (21.9%)	52 (26.5%)	3	3.44	1.24
5. I was satisfied with my remote course work.	23 (11.7%)	28 (14.3%)	36 (18.4%)	46 (23.5%)	63 (32.1%)	4	3.5	1.38
*Item*	*Strongly disagree Code = 5*	*Disagree Code = 4*	*Neutral Code = 3*	*Agree Code = 2*	*Strongly agree Code = 1*			
6. I cheated during my remote courses.*	97 (49.5%)	24 (12.2%)	40 (20.4%)	22 (11.2%)	13 (6.6%)	2	2.13	1.32
*Item*	*Strongly disagree Code = 1*	*Disagree Code = 2*	*Neutral Code = 3*	*Agree Code = 4*	*Strongly agree Code = 5*			
7. My professors used effective instructional strategies during my remote course work.	13 (6.6%)	33 (16.8%)	55 (28.1%)	41 (20.9%)	54 (27.6%)	3	3.46	1.24
8. My professors used effective assessment strategies during my remote course work.	11 (5.6%)	27 (13.8%)	59 (30.1%)	46 (23.5%)	53 (27.0%)	4	3.52	1.19
9. My professors were engaged for my remote course work.	11 (5.6%)	22 (11.2%)	60 (30.6%)	36 (18.4%)	67 (34.2%)	4	3.64	1.21

COAMS, College of Applied Medical Sciences; Mdn, Median; μ, Mean; and SD, Standard deviation. $\text{Percentage of Responses}\;\left(\%\right)=\frac{\text{Number of Responses}}{196}\times 100.$ ^*^Items where agreement indicated negative behavior, such as cheating, were reverse coded for the purposes of reliability analysis.

**Table 5:** COAMS students’ initial perceptions about remote learning.

Item	Strongly disagree Code = 1	Disagree Code = 2	Neutral Code = 3	Agree Code = 4	Strongly agree Code = 5	Summary statistics		
	*n* (%)					Mdn	μ	SD
1. My internet access was adequate for remote instruction.	7 (3.6%)	14 (7.1%)	47 (24.0%)	51 (26.0%)	77 (39.3%)	4	3.90	1.11
2. My internet speed was adequate for remote instruction.	5 (2.6%)	20 (10.2%)	42 (21.4%)	62 (31.6%)	67 (34.2%)	4	3.85	1.08
3. The instructional methods used by my professors translated to remote instruction.	3 (1.5%)	16 (8.2%)	62 (31.6%)	67 (34.2%)	48 (24.5%)	4	3.72	0.98
4. The assessment methods used by my professors translated to remote instruction.	4 (2.0%)	20 (10.2%)	66 (33.7%)	53 (27.0%)	53 (27.0%)	4	3.67	1.05
5. My professors worked harder to provide remote instruction than on-site instruction.	14 (7.1%)	25 (12.8%)	77 (39.3%)	39 (19.9%)	41 (20.9%)	3	3.35	1.16
6. My professors worked harder to provide on-site instruction than remote instruction.	13 (6.6%)	14 (7.1%)	70 (35.7%)	54 (27.6%)	45 (23.0%)	4	3.53	1.12
7. I was satisfied with my remote instruction.	21 (10.7%)	26 (13.3%)	50 (25.5%)	38 (19.4%)	61 (31.1%)	4	3.47	1.34

COAMS, College of Applied Medical Sciences, Mdn, Median; μ, Mean; and SD, Standard deviation. $\text{Percentage of Responses}\;\left(\%\right)=\frac{\text{Number of Responses}}{196}\times 100.$

**Table 6:** COAMS students’ perceptions of their effort and engagement.

Item	Strongly disagree Code = 1	Disagree Code = 2	Neutral Code = 3	Agree Code = 4	Strongly agree Code = 5	Summary statistics		
	*n* (%)					Mdn	μ	SD
1. I tried my best during remote instruction.	12 (6.1%)	9 (4.6%)	33 (16.8%)	48 (24.5%)	94 (48.0%)	4	4.04	1.18
2. My classmates tried their best during remote instruction.	4 (2.0%)	15 (7.7%)	48 (24.5%)	48 (24.5%)	81 (41.3%)	4	3.95	1.07
3. I attended remote classes.	7 (3.6%)	14 (7.1%)	29 (14.8%)	47 (24.0%)	99 (50.5%)	5	4.11	1.12
4. I actively engaged in remote classes.	11 (5.6%)	32 (16.3%)	46 (23.5%)	54 (27.6%)	53 (27.0%)	4	3.54	1.21
5. I felt accountable to myself to complete remote coursework.	9 (4.6%)	15 (7.7%)	49 (25.0%)	58 (29.6%)	65 (33.2%)	4	3.79	1.12
6. I felt accountable to my professors to complete remote coursework.	8 (4.1%)	18 (9.2%)	54 (27.6%)	60 (30.6%)	56 (28.6%)	4	3.70	1.10
7. I marked myself as attended but did NOT participate in my remote course work.	45 (23.0%)	33 (16.8%)	45 (23.0%)	45 (23.0%)	28 (14.3%)	3	2.89	1.37
8. My professors tried their best during remote instruction.	6 (3.1%)	18 (9.2%)	49 (25.0%)	47 (24.0%)	76 (38.8%)	4	3.86	1.13

COAMS, College of Applied Medical Sciences; Mdn, Median; μ, Mean; and SD, Standard deviation. $\text{Percentage of Responses}\;\left(\%\right)=\frac{\text{Number of Responses}}{196}\times 100.$

**Table 7:** COAMS students’ perceptions concerning their needs, behavior, and effort.

Item	Strongly disagree Code = 1	Disagree Code = 2	Neutral Code = 3	Agree Code = 4	Strongly agree Code = 5	Summary statistics		
	*n* (%)					Mdn	μ	SD
1. My individual needs were met through remote instruction.	6 (3.1%)	31 (15.8%)	62 (31.6%)	49 (25.0%)	48 (24.5%)	3	3.52	1.12
2. I contacted at least one professor independently during remote instruction.	20 (10.2%)	18 (9.2%)	55 (28.1%)	50 (25.5%)	53 (27.0%)	4	3.5	1.26
3. I worked harder than my professors during remote instruction.	15 (7.7%)	35 (17.9%)	76 (38.8%)	35 (17.9%)	35 (17.9%)	3	3.2	1.16
4. My professors worked harder than me during remote instruction.	12 (6.1%)	33 (16.8%)	85 (43.4%)	40 (20.4%)	26 (13.3%)	3	3.12	1.06

COAMS, College of Applied Medical Sciences; MdnMedian; μ, Mean; and SD, Standard deviation. $\text{Percentage of Responses}\;\left(\%\right)=\frac{\text{Number of Responses}}{196}\times 100.$

**Table 8:** COAMS students’ ethical behavior.

	*n* (%)					Mdn	μ	SD
Item	Strongly disagree Code = 1	Disagree Code = 2	Neutral Code = 3	Agree Code = 4	Strongly agree Code = 5	Summary statistics		
1. I collaborated with classmates to complete individually assigned remote course work.	28 (14.3%)	20 (10.2%)	40 (20.4%)	58 (29.6%)	50 (25.5%)	4	3.42	1.35
2. I collaborated with people not in my classes to complete individually assigned remote course work.	69 (35.2%)	20 (10.2%)	36 (18.4%)	42 (21.4%)	29 (14.8%)	3	2.70	1.49
3. I collaborated with classmates to complete tests in remote classes.	55 (28.1%)	24 (12.2%)	48 (24.5%)	32 (16.3%)	37 (18.9%)	3	2.86	1.47
*Item*	*Strongly disagree Code = 5*	*Disagree Code = 4*	*Neutral Code = 3*	*Agree Code = 2*	*Strongly agree Code = 1*			
4. Per my professors’ definitions of cheating, I cheated at least once in remote classes.*	62 (31.6%)	23 (11.7%)	39 (19.9%)	43 (21.9%)	29 (14.8%)	3	2.76	1.47
5. Per my definition of cheating, I cheated at least once in remote classes.*	62 (31.6%)	27 (13.8%)	45 (23.0%)	39 (19.9%)	23 (11.7%)	3	2.67	1.40

COAMS, College of Applied Medical Sciences; Mdn, Median; μ, Mean; and SD, Standard deviation. $\text{Percentage of Responses}\;\left(\%\right)=\frac{\text{Number of Responses}}{196}\times 100.$ ^*^Items where agreement indicated negative behavior, such as cheating, were reverse coded for the purposes of reliability analysis.

**Table 9:** COAMS students’ general perceptions of “Hybrid” learning.

Item	Strongly disagree Code = 1	Disagree Code = 2	Neutral Code = 3	Agree Code = 4	Strongly agree Code = 5	Summary statistics		
	*n* (%)					Mdn	μ	SD
1. My assessment scores better with hybrid learning.	20 (10.2%)	18 (9.2%)	60 (30.6%)	41 (20.9%)	57 (29.1%)	3.5	3.5	1.27
2. I think hybrid learning prepared me well to embark on treating patients.	21 (10.7%)	28 (14.3%)	60 (30.6%)	36 (18.4%)	51 (26.0%)	3	3.35	1.29
3. Hybrid course is effective to reach designated learning objectives.	11 (5.6%)	21 (10.7%)	64 (32.7%)	46 (23.5%)	54 (27.6%)	4	3.57	0.08
4. I believe hybrid education will improve my health knowledge and health skills.	13 (6.6%)	28 (14.3%)	56 (28.6%)	50 (25.5%)	49 (25.0%)	4	3.48	1.2
5. Future health education in combined courses (theory and clinical/practical) may be hybrid.	16 (8.2%)	22 (11.2%)	47 (24.0%)	50 (25.5%)	61 (31.1%)	4	3.60	1.26
6. I feel more confident with hybrid learning.	18 (9.2%)	21 (10.7%)	55 (28.1%)	46 (23.5%)	56 (28.6%)	4	3.52	1.26

COAMS, College of Applied Medical Sciences; Mdn, Median; μ, Mean; and SD, Standard deviation. $\text{Percentage of Responses}\;\left(\%\right)=\frac{\text{Number of Responses}}{196}\times 100$.

The results presented in Table 6 are specifically aimed at addressing the effort and engagement of COAMS students while taking online classes. Most of COAMS students agreed and strongly agreed that they tried their best (*n* = 142, 72.5%) and that their professors also tried their best (*n* = 123, 62.8%) during remote instruction. Likewise, a majority of COAMS students attended remote classes (*n* = 146, 74.5%), while 54.6% (*n* = 107) agreed and strongly agreed that they actively engaged in remote classes. Additionally, a large percentage of COAMS students agreed and strongly agreed that they felt accountable to both themselves (*n* = 123, 62.8%) and their professors (*n* = 116, 59.2%) to complete remote coursework. Moreover, 39.8% of COAMS students (*n* = 78) disagreed and strongly disagreed with the statement that they marked themselves as attending but did not participate in their remote coursework; 37.3% (*n* = 73) agreed and strongly agreed, and 23% (*n* = 45) were neutral.

Table 7 presents results regarding the behavior and needs of COAMS students in taking online classes and their perceived effort as compared to their instructors. Nearly half of COAMS students (*n* = 97, 49.5%) have agreed and strongly agreed that remote instruction met their individual needs. Likewise, 52.5% of COAMS students (*n* = 103) have communicated independently with at least one professor during remote instruction. Moreover, 35.5% of COAMS students (*n* = 70) have agreed and strongly agreed that they worked harder than their professors during remote instruction; 25.6% (*n* = 50) disagreed and strongly disagreed, and 38.8% (*n* = 76) were neutral. On the other hand, 33.7% of COAMS students (*n* = 66) have agreed and strongly agreed that their professors worked harder than them during remote instruction; whereas 22.9% (*n* = 45) disagreed and strongly disagreed, and 43.4% (*n* = 85) were neutral.

Table 8 provides results regarding the ethical behavior of COAMS students during remote instruction. Over half of COAMS students (*n* = 108, 55.0%) have agreed and strongly agreed that they collaborated with classmates to complete individually assigned remote coursework, while 36.2% (*n* = 71) collaborated with people not in their classes, and 35.2% (*n* = 69) collaborated with classmates to complete tests in remote classes. Additionally, 36.7% of COAMS students (*n* = 72) have agreed and strongly agreed that they cheated at least once in remote classes as defined by their professors; whereas 43.3% (*n* = 85) disagreed and strongly disagreed, and 19.9% (*n* = 39) were neutral. Similarly, 31.6% of COAMS students (*n* = 62) have agreed and strongly agreed that they cheated at least once in remote classes as per their definitions of cheating; whereas 45.4% (n = 89) disagreed and strongly disagreed, and 23.0% (*n* = 45) were neutral.

Table 9 shows results regarding the general perception of “Hybrid” learning among COAMS students. Approximately half of COAMS students have agreed and strongly agreed that a hybrid learning is applicable for future health education (*n* = 111, 56.6%), effective to reach designated learning objectives (*n* = 100, 51.1%), will improve health knowledge and health skills (*n* = 99, 50.5%), may enable better assessment scores (*n* = 98, 50.0%), may enable more confidence among students (*n* = 102, 52.1%). Furthermore, 44.4% of COAMS students (*n* = 87) have agreed and strongly agreed that hybrid learning prepare students well to embark on treating patients.; whereas 25.0% (*n* = 49) disagreed and strongly disagreed, and 30.6% (*n* = 60) were neutral.

### Questionnaire findings by demographic variables

Table 10 presents a summary of the findings from the analysis of demographic variables, which includes gender (i.e., female versus male) and academic year (i.e., 4th versus 3rd). To satisfy the assumptions required for the Chi-Square tests in the current study, it was necessary to merge the agree and strongly agree categories, as well as the disagree and strongly disagree categories. Therefore, the Chi-Square tests were conducted based on three response categories, namely agree, neutral, and disagree.

**Table 10:** Differences in percentages across COAMS Students’ gender and academic year.

Dimension / Subscale	Item	Responses	Variable		$\chi^{2}$	*p*
			Academic year “*n* (%)”			
General Perceptions of “Remote” Learning prior to COVID-19	Item 6- My professors used effective instructional strategies during my remote course work		3rd year *n* = 106	4th year *n* = 90		
		Disagree	20 (18.9%)	26 (28.9%)	6.176	0.046
		Neutral	37 (34.9%)	18 (20%)		
		Agree	49 (46.2%)	46 (51.1%)		
			Gender “*n* (%)”			
	Item 8- My professors were engaged for my remote course work.		Female *n* = 102	Male *n* = 94		
		Disagree	16 (15.7%)	17 (18.1%)	11.507	0.003
		Neutral	42 (41.2%)	18 (19.1%)		
		Agree	44 (43.1%)	59 (62.8%)		
	Item 9- I was satisfied with my remote course work.	Disagree	31 (30.4%)	20 (21.3%)	8.124	0.017
		Natural	24 (23.5%)	12 (12.8%)		
		Agree	47 (46.1%)	62 (65.9%)		
Initial Perceptions about Remote Learning	Item 7- I was satisfied with my remote instruction.	Disagree	28 (27.5%)	19 (20.2%)	9.452	0.009
		Neutral	33 (32.3%)	17 (18.1)		
		Agree	41 (40.2%)	58 (61.7%)		
Perceptions concerning COAMS Students’ needs, behavior, and effort	Item 1- My individual needs were met through remote instruction.	Disagree	24 (23.53%)	13 (13.83%)	9.100	0.011
		Neutral	38 (37.25%)	24 (25.53%)		
		Agree	40 (39.22%)	57 (60.64%)		
	Item 4- My professors worked harder than me during remote instruction.	Disagree	30 (29.4%)	15 (16%)	8.233	0.016
		Neutral	46 (45.1%)	39 (41.5%)		
		Agree	26 (25.5%)	40 (42.5%)		

COAMS, College of Applied Medical Sciences; *χ*^2^, Chi Square; *p* = *p*-value. $\text{Percentage of Responses}\;\left(\%\right)=\frac{\text{Number of Responses}}{\text{Total number of students in each catogorical variable}}\times 100$.

There was a statistically significant difference between 3rd and 4th year students (*p* = 0.046) in the dimension/subscale pertaining to the general perceptions of “Remote” learning prior to COVID-19. A total of 37 (34.9%) of 3rd year students remained neutral when asked if their professors used effective instructional strategies during remote coursework, compared to 18 (20%) of 4th year students. There were no statistically significant differences between 3rd and 4th year students in the other dimensions/subscales.

There was a statistically significant difference between male and female students in the dimension/subscale pertaining to the general perceptions of “Remote” learning prior to COVID-19. A total of 59 (62.8%) male students agreed as to whether their professors were engaged during remote coursework, compared to 44 (43.1%) female students (*p* = 0.003). Moreover, 62 (65.9%) of male students were satisfied with remote coursework, compared to 47 (46.1%) female students (*p* = 0.017). There was a statistically significant difference between male and female students (*p* = 0.009) in the dimension/subscale pertaining to the initial perceptions about “Remote” learning. A total of 58 (61.7%) male students were satisfied with remote instruction, compared to 41 (40.2%) female students. There was a statistically significant difference between male and female students in the dimension/subscale pertaining to the perceptions concerning COAMS students’ needs, behavior, and effort. A total of 57 (60.64%) male students agreed that their individual needs were met through remote instruction, compared to 40 (39.22%) female students (*p* = 0.011). Moreover, 40 (42.5%) of male students agreed that their professors worked harder than them during remote instruction, compared to 26 (25.5%) female students (*p* = 0.016). There were no statistically significant differences between male and female students in the other dimensions/subscales.

## Discussion

This study offers valuable insight into the perceptions of COAMS students regarding their transition from in-person to remote and hybrid learning because of COVID-19 pandemic. To our knowledge, this is the first study to explore perceptions and experiences of COAMS students regarding in-person versus remote or hybrid learning in Saudi Arabia. Over 70% of COAMS students were satisfied and engaged in their on-site coursework. However, based on data obtained from questionnaires, it appears that their satisfaction and level of engagement diminished following their shift to remote learning. Similarly, more than 62% of COAMS students were satisfied with their instructors’ use of effective instructional and assessment strategies and level of engagement during on-site coursework; and such perceptions have decreased with remote instruction. Furthermore, the results of the questionnaire suggest that hybrid learning can be beneficial and effective in improving the performance and learning experience of COAMS students. The COAMS male students were more satisfied with remote learning because it met their needs and they were more satisfied with their instructors’ performance during remote instruction, compared to female students.

The importance of face-to-face instruction in medical education and applied health sciences has long been acknowledged. However, the old paradigm has been challenged by rising clinical demands and time constraints. Over the past few decades, there has been a discernible movement toward online, remote, or electronic learning in medical education. As a result of this change, medical educators need to adjust to shifting conditions and develop instructional strategies that are consistent with the changing needs of the applied health profession (22).

The survey results from this study showed that while switching to remote instruction during the Spring 2020 semester, students’ initial favourable views and satisfaction with their teaching began to diminish. For some educators, offering effective instruction may be difficult given this shift in views. Significant obstacles must be overcome for remote e-learning to be used in applied health education, especially in low- and middle-income nations. There are three basic categories of impediments to implementing remote e-learning: institutional/educator barriers, student barriers, and technology/infrastructure barriers. These obstacles, which impact both students and faculty members, include a lack of infrastructure, restricted access to technology and internet services, and poor internet quality (57, 58). The problems reported by the participants in this study, as outlined in Tables 4, 5, reflected these issues. Participants who reside in rural locations may have been impacted more by these impediments.

COAMS students in the study showed great accountability to themselves (62.8%) and their professors (59.2%) for completing remote coursework. It may be challenging for students who struggle with self-regulation of their work pace to take part in online learning courses, especially fully asynchronous courses. Participants in the study acknowledged being engaged, motivated, and felt accountable during remote instruction. Similarly, studies reported that the sudden transition to remote instruction led to overwhelming acceptance by students. There was adequate flexibility from instructors, and students appreciated the sense of engagement they typically experienced in their in-person classes (6, 59). However, some studies report that students were less engaged, less motivated, and less accountable after switching to remote learning (6, 50, 52).

Vast majority of the study participants disagreed to cheating during on-site courses (81.1%) and remote courses (61.7%). As previously mentioned, participants in this study felt engaged and felt accountable. However, previous studies report that faculty and students experienced significant challenges when switching to online or remote learning due to abrupt changes and limited planning time. Students compensate for their lack of focus and engagement by collaborating on assignments, such as tests and quizzes. Their actions were justified by feelings of overwhelm and a desire to finish the semester. Cheating was often perceived as a means to an end in their pursuit of academic progress (12, 59).

In this study, most students expressed favourable opinions on hybrid learning. Similarly, Raes et al. found that flexible hybrid learning environments increased attendance among students who otherwise took sick days or faced home-related problems. The two modes of contact offered by this strategy also made it easier for underrepresented groups to equalize their learning opportunities and offered more thorough support (60). However, some students may feel less connected to the teacher and one another because of the hybrid class structure, and in many cases, active class participation is challenging (53). While the COVID-19 outbreak brought attention to educational inequality, most study participants had access to the internet and enough bandwidth for distance learning. The instructors’ efforts to foster a conducive learning environment may have led to student responsibility being greater than expected.

Effective teaching is the key to success in an emergency remote teaching environment such as during the COVID-19 Pandemic (25). The characteristics of the instructor play a vital role in enhancing student engagement and satisfaction. It is imperative that the instructor provides cognitive and affective support to students during times of substantial change, with clear communication being also crucial in ensuring students receive the best possible education (25). Previous studies have shown that student satisfaction in stable learning environments is largely determined by the instructor’s ability to communicate clearly, provide clear explanations, and provide appropriate learning materials, all of which are cognitive factors (61–64). However, these aspects become more important during periods of significant change, such as the COVID-19 pandemic (25). While student engagement can be affected by changes in the learning environment during stable periods, it was even more crucial in a crisis learning environment where students urgently needed to devote significant time and effort to their learning in order to cope with the sudden changes imposed on them (25).

The study instrument used in this research contained items that measured the three dimensions of student engagement in various learning environments, including on-site, remote, and hybrid classes. Various indicators were used to measure the behavioral engagement of COAMS students prior and during COVID-19 including attendance, coursework completion, getting involved, study habits, achievement, and confidence as shown in Tables 3, 6, 8, 9. A larger proportion of COAMS students (77.1%) indicated that they attended on-site classes while 74.5% attended remote classes. The percentage of COAMS students who indicated that they completed on-site coursework were 77.6%, while 59.2–62.8% felt accountable to both themselves and their professors to complete remote coursework. Additionally, 64.8% of COAMS students indicated that they were engaged in on-site courses and 54.6% engaged in remote courses. A larger proportion of COAMS students (81.1%) indicated that they did not cheat during on-site courses, while 45.4% not cheating once during remote courses. Furthermore, 50% of COAMS students indicated that hybrid learning enables better assessment scores, and 52.1% indicated that hybrid learning may enable more confidence. On the other hand, behavioral disengagement was also measured as 39.8% of COAMS students indicated that they marked themselves as attending but did not participate in their remote coursework during to COVID-19 (Table 6).

Affective engagement was also measured through indicators related to COAMS students’ positive emotions including attitude, satisfaction, and interaction in response to the teacher, peers, and the education environment as shown in Tables 3, 5–7, 9. The majority of COAMS students (70.4%) were satisfied with on-site coursework, while 50.5% were satisfied with remote instruction. Most COAMS students indicated that their professors used effective instructional strategies during on-site coursework (62.8%), used effective assessment strategies (64.8%), and engaged them in the on-site instruction (71.9%). More than half of the COAMS students indicated that the instructors’ instructional methods (58.7%), and assessment methods (54%) translated well into remote instruction. Also, 65.5% of COAMS students indicated that internet access and internet speed were adequate for remote instruction, 62.8–65.8% indicated that their classmates and their professors tried their best during remote instruction, 40.8% indicated that their professors worked harder to provide remote instruction than on-site instruction, while 50.6% indicated that their professors worked harder to provide on-site instruction than remote instruction. Additionally, nearly half of COAMS students (49.5%) indicated that remote instruction met their individual needs, and 33.7% indicated that their professors worked harder than them during remote instruction. As for hybrid learning environment, 56.6% of COAMS students indicated that hybrid learning is applicable for future health education, 51.1% indicated it would improve health knowledge and skills, and 44.4% indicated it would prepare students well to embark on treating patients in the future.

Cognitive engagement was also measured through indicators related to COAMS students’ ability to actively participate in self-regulated learning, including the perceived relevance of material to their experience, positive self-perception, and learning from peers as shown in Tables 3, 5–9. Most COAMS students indicated that their professors used effective instructional strategies during on-site coursework (62.8%), and used effective assessment strategies (64.8%). More than half of the COAMS students indicated that the instructors’ instructional methods (58.7%), and assessment methods (54%) translated well into remote instruction. Most COAMS students (72.5%) indicated that they tried their best during remote instruction. Additionally, 52.5% COAMS students have communicated independently with at least one professor, 55% have collaborated with classmates to complete individually assigned remote coursework, while 36.2% collaborated with people not in their classes, and 35.2% collaborated with classmates to complete tests in remote classes. Furthermore, 56.6% of COAMS students indicated that a hybrid course is effective to reach designated learning objectives. On the other hand, cognitive disengagement was also measured by indicators related to feeling pressured or challenged or overwhelmed as 36.2% of COAMS students indicated that they were challenged by on-site coursework, and 40.3% were challenged by remote coursework (Tables 3, 4).

In our study, we found that students’ satisfaction levels as a measure or an indicator of affective/emotional engagement declined following the shift to remote learning. This finding is consistent with a recent study that reported a decreasing level of emotional engagement among 73 undergraduate science students at 23 different colleges and universities across the United States, as well as a drastic decline in students’ positive attitudes toward science (65). Students’ engagement is essential as a significant driver of their motivation, self-directed learning, ability to retain information, overall well-being, and other factors related to their academic success (65, 66). As students transitioned to distance learning setting, they encountered a limited number of options for a stable and established learning environment. Additionally, the stress they experienced as a result of the abrupt transition may have affected their memory and learning (67, 68). By recognizing that students’ cognitive engagement becomes stagnant with a decline in emotional engagement, we can enhance our ability to design effective mechanisms to foster student learning amidst education disruptions brought on by emergencies (65). In addition to students’ engagement, instructors and leaders of educational institutions should continue to address the concerns reported about remote teaching, including its effectiveness, quality, costs, and equity (69).

As a result of the COVID-19 outbreak, the field of interprofessional health education has embraced remote learning as its primary mode of delivery in order to effectively engage students and facilitate the acquisition of knowledge and skills. Nonetheless, it is important to recognize that learning is not solely focused on the acquisition of knowledge and skills, but rather is a vital endeavor that contributes to transformation and enrichment of interprofessional health sciences students (70). As remote and hybrid learning is expected to become an integral part of interprofessional health education in the future, it is essential for interprofessional health education researchers to gather not only the perspectives of faculty and staff regarding the transition and their experience but also those of students. The importance of this is even greater for COAMS students, who come from eight applied health sciences programs/professions, including Anesthesia Technology, Clinical Laboratory Science, Clinical Nutrition, Echocardiography Cardiovascular Technology, Emergency Medical Services, Occupational Therapy, Radiological Sciences, and Respiratory Therapy. The curriculum of COAMS programs offers exceptional interprofessional undergraduate education with a thorough approach that integrates intensive theoretical teaching and practical training to maximize the students’ full potential. Although the COAMS programs follow traditional instructional methods, the curriculum also fosters an interactive and cooperative learning environment through the integration of diverse teaching strategies such as problem-based learning (PBL), small group discussions, and simulation to ensure achieving the learning outcomes. Challenges to remote interprofessional health education may include issues related to communication and barriers related to educational content and interaction between instructor and student, inadequacy for practical learning, student assessment, use of technology tools, online experience and inadequacy of internet and website services, pandemic-related anxiety or stress or burnout, time management, lack of motivation and technophobia (2, 3, 71, 72). Besides the findings of our study, these challenges indicate the areas that require attention in interventions aimed at facilitating the successful implementation of interprofessional online education challenges, based on COAMS students’ perspectives in Saudi Arabia and elsewhere.

### Study limitations

Using non-probability convenience sampling to gather data limits the study’s generalizability, because it does not adequately reflect the community. Furthermore, the focus of this study was a single college in the KSA, which makes it difficult for the results to be generalized. Further research across several Saudi universities will be necessary to generalize the findings and provide a more comprehensive picture of COAMS students’ perspectives and attitudes towards remote and hybrid learning. Moreover, the lower sample size of COAMS 4th and 3rd year students enrolled in some academic programs may have limited inter-program comparisons.

## Conclusion

This study sheds light on the perceptions of undergraduate COAMS Saudi students about the change from on-site to remote/hybrid learning during the COVID-19 pandemic. The findings show that remote classrooms have lower attendance and interest than on-site classes, and cheating incidents have increased. Despite lower satisfaction levels in online courses, hybrid learning was viewed favourably by COAMS students. In contrast to female students, COAMS male students were more satisfied with remote instruction because it met their needs. In light of these findings, plans should be developed to increase student involvement, improve academic integrity, and assess the effect of the pandemic on undergraduate education on a regular basis. By incorporating these measures, educational institutions can enhance and support the remote learning experience for their students. Based on the findings, several suggestions are offered to enhance student satisfaction, enhance distance learning experiences, and alleviate the difficulties caused by the epidemic. Among them are methods to raise student participation, deal with academic dishonesty, provide sufficient support services, improve instructor communication and training, investigate the potential of hybrid learning, and continuously assess the pandemic’s impact on undergraduate students. Putting these tips into practice can make remote learning programs more effective and efficient at higher educational institutions.

## Data availability statement

The raw data supporting the conclusions of this article will be made available by the authors, without undue reservation.

## Ethics statement

King Abdullah International Medical Research Center ethics committee approved this study (Study Number: SP22J/123/08). We confirm that a written informed consent was obtained from the study participants and that the guidelines outlined in the Declaration of Helsinki were followed.

## Author contributions

KA: Conceptualization, Data curation, Investigation, Methodology, Resources, Software, Supervision, Validation, Visualization, Writing – original draft, Writing – review & editing. EG: Data curation, Formal analysis, Methodology, Project administration, Resources, Software, Supervision, Writing – review & editing. MarA: Data curation, Formal analysis, Investigation, Methodology, Project administration, Software, Writing – original draft. HA: Data curation, Formal analysis, Investigation, Methodology, Project administration, Software, Writing – original draft. MoA: Data curation, Formal analysis, Investigation, Methodology, Project administration, Software, Writing – original draft. ZA: Investigation, Methodology, Resources, Software, Supervision, Validation, Visualization, Writing – review & editing. MajA: Investigation, Methodology, Project administration, Resources, Supervision, Visualization, Writing – review & editing.

## Funding

The author(s) declare that no financial support was received for the research, authorship, and/or publication of this article.

## Conflict of interest

The authors declare that the research was conducted in the absence of any commercial or financial relationships that could be construed as a potential conflict of interest.

## Publisher’s note

All claims expressed in this article are solely those of the authors and do not necessarily represent those of their affiliated organizations, or those of the publisher, the editors and the reviewers. Any product that may be evaluated in this article, or claim that may be made by its manufacturer, is not guaranteed or endorsed by the publisher.
